# The generative potential of mess in community-based participatory research with young people who use(d) drugs in Vancouver

**DOI:** 10.1186/s12954-022-00615-7

**Published:** 2022-03-25

**Authors:** Madison Thulien, Haleigh Anderson, Shane Douglas, Rainbow Dykeman, Amanda Horne, Ben Howard, Kali Sedgemore, Reith Charlesworth, Danya Fast

**Affiliations:** 1 British Columbia Centre on Substance Use, 1045 Howe St Suite 400, Vancouver, BC V6Z 2A9 Canada; 2 Youth Health Advisory Council, Vancouver, Canada; 3 Coalition of Peers Dismantling the Drug War, Vancouver, Canada; 4 Department of Medicine, University of British Columbia, Vancouver, Canada

**Keywords:** Community-based participatory research, Qualitative health research, Young people, Substance use, Ethics

## Abstract

Community-based participatory research (CBPR) is increasingly standard practice for critical qualitative health research with young people who use(d) drugs in Vancouver, Canada. One aim of CBPR in this context is to redress the essentialization, erasure, and exploitation of people who use(d) drugs in health research. In this paper, we reflect on a partnership that began in 2018 between three university researchers and roughly ten young people (ages 17–28) who have current or past experience with drug use and homelessness in Greater Vancouver. We focus on moments when our guiding principles of shared leadership, safety, and inclusion became fraught in practice, forcing us in some cases to re-imagine these principles, and in others to accept that certain ethical dilemmas in research can never be fully resolved. We argue that this messiness can be traced to the complex and diverse positionalities of each person on our team, including young people. As such, creating space for mess was ethically necessary and empirically valuable for our CBPR project.

## Introduction

In this article, we tell a story about our research team. At the time of writing, we were comprised of three university researchers (Madison, Reith, and Danya) and a youth^1^ advisory council (YAC) of ten young people (ages 17–28) with current or past experience with drug use and homelessness in Greater Vancouver (see Table 1).^2^ Conducting research through collaborative partnerships like ours is the defining feature of community-based participatory research (CBPR; [10, 34, 64]). In short, CBPR represents a shift in approach from research *on* to research *with* individuals and groups.

**Table 1:** Team members’ chosen demographic identifiers for this article

Name (YAC members' names are pseudonyms)	Chosen demographic identifiers
Kendra	White woman, pansexual, harm reduction activist
Ocean	Woman who doesn't identify with specific pronouns or a single ethnicity, fallen through every crack in ‘the system,’ fighting to make the world a better place
Alanna	*We lost touch with Alanna after she moved back to her parents’ home in the winter of 2019*
Jordan	*We lost touch with Jordan after he left Vancouver in the winter of 2018*
Thorn	Indigenous person, IV meth user, harm reduction and youth drug user activist, peer worker
Raven	Cree Métis, Two-spirit person, former foster kid
Stan	White male, gay, demon, bold and unafraid to stand out
Kat	Métis woman, person in recovery, loved by family
Madison	White settler of European and South Asian heritage, cis woman, ally
Reith	White settler, cis woman, ally and activist
Danya	White settler, cis woman, mother, university professor

CBPR is applied in various disciplines, and is increasingly standard practice for critical qualitative health research (i.e., qualitative research that attends to the power dynamics and social positions that shape health-related phenomena [16]). In a hyper-researched setting like Vancouver, we are often asked about our experiences conducting CBPR with young people who use(d) drugs. In short, our partnership has deeply enriched our work and led to novel accomplishments. It has also been punctuated by messy moments, particularly surrounding our guiding principles of shared leadership, safety, and inclusion. Despite our shared commitments to these principles, their application has at times been fraught—forcing us in some cases to re-imagine them, and in others to accept that certain ethical dilemmas in research can never be fully resolved.

We follow others in our use of the term ‘mess’ to characterize difficult—although important—moments in CBPR [12, 37, 58]. By mess, we are referring to the contradictions, tensions, misunderstandings, and complexities that characterized certain encounters [20]. In these moments, our various commitments and priorities became entangled, and the best way forward was unclear. We argue that the messiness surrounding our guiding principles can be traced to the complex and diverse positionalities of each person on our team, including young people. As such, creating space for mess was ethically necessary and empirically valuable for our CBPR project.

We begin by reviewing the CBPR literature related to young people and substance use. After describing our methods for creating this article, we introduce our research team and describe the setting of our YAC meetings. Finally, we present ethnographic vignettes detailing the messiness surrounding our guiding principles, and the insights generated in those encounters. Our conclusions are of relevance to others conducting CBPR in contexts where shared leadership, safety, and inclusion are essential principles of ethical CBPR, and who find themselves navigating confusing, complicated, and messy research encounters.

## Research with young people who use(d) drugs

Our team’s work occurs at the intersection of youth health and substance use research, and recognizes their parallel legacies of essentialization, erasure, exploitation, and extraction. Health research has traditionally essentialized youth and people who use(d) drugs as ‘problems’ to be solved and ‘vulnerable populations’ in need of saving [17, 50, 56]. This framing is based on paternalistic assumptions that younger age and drug use render individuals incapable of autonomy and self-determination [17, 50]. Because young people who use(d) drugs occupy the intersection of these stigmas, their voices, lived realities, and priorities are often erased from health research [47]. Traditionally and continuing into the present, health research has also been largely extractive: researchers enter communities of people who use(d) drugs, including youth, to take knowledge that can be used to publish papers, secure further grant funding, and build careers [18]. Meanwhile, for the communities under study, research often does not result in solutions, funding, or ownership of the ideas generated [18].

In an effort to address these legacies of essentialization, erasure, exploitation, and extraction, health researchers like ourselves are increasingly turning to CBPR [64]. The roots of CBPR can be traced to the emancipatory pedagogy of Brazilian educator Paulo Freire [25], Indigenous sovereignty movements globally [10], and ethical frameworks advanced by Indigenous and feminist scholars [40, 63]. Participatory approaches gained international momentum in the early 1990s. The 1989 United Nations Declaration on the Rights of the Child, ratified by Canada in 1991, states that young people have the right to be involved in research [60]. Internationally, disability rights activists began organizing around the phrase ‘nothing about us without us’ to demand their inclusion in research and policy-making that shapes their lives [11]. This phrase has since been taken up by other activist groups around the world, including people who use(d) drugs [4, 33, 36].

These global movements have bolstered local activism in Vancouver. In our province of British Columbia, various government agencies undertook participatory projects with youth in the 1990s and 2000s, including the creation of a Ministry of Child and Family Development (MCFD) Youth Advisory Council and the City of Vancouver’s Civic Youth Strategy [28, 30]. In addition, the persistent efforts of activist groups like the Vancouver Area Network of Drug Users (VANDU) established CBPR as an important modality for research involving people who use(d) drugs [41, 61], including youth who use(d) drugs (see for example, [13, 26, 44]).

The form and function of CBPR partnerships can vary widely depending on the goals, needs, and circumstances of those involved [52]. In our case, we decided to establish an ‘advisory council’ composed of young stakeholders who would provide guidance and participate in decision-making. This model was chosen because it allows for flexible participation; members of an advisory council can skip meetings if they need to, and can say as much or as little as is comfortable for them during meetings [52]. The advisory council model has been applied successfully in CBPR studies with people who use(d) drugs [39, 57]. Additionally, we chose a YAC because it is a common participatory model locally; other examples include  the aforementioned MCFD YAC [30], the Vancouver Aboriginal Child and Family Services Society YAC [1], and the McCreary Centre Society YAC [54].

As CBPR has gained popularity in Vancouver and elsewhere, a number of practitioners have reflected critically on the ethics of collaborative research [43], including with youth [8]. Some have highlighted the ‘naïve or inadequate assumptions’ ([53], p. 169) that can exist at the outset of a project, which then break down in practice [32, 42, 58]. Similarly, substance use researchers have troubled the assumption that CBPR is an inherently more ethical form of research, demonstrating how CBPR can stigmatize, exploit, and over-burden people who use(d) drugs [15, 18, 23, 55, 57]. Drug user activists in Vancouver have called out ‘tokenism’ in some participatory projects, referring to projects that involve people who use(d) drugs superficially but deny them meaningful control or influence [45, 65]. Scholars have raised similar concerns about tokenism within youth-focused CBPR projects [3, 8]. Collectively, these critiques call on us to continuously reflect on the ethics of our research and make adjustments when our assumptions about how to do CBPR are challenged.

In this article, we join others in advancing the critical literature on CBPR, and in particular CBPR involving young people who use(d) drugs in the context of entrenched poverty and homelessness. To date, there have been relatively few CBPR studies focused on youth and substance use. Those studies that have been undertaken typically exclude youth who are actively using drugs at the time of their participation [13, 15, 23, 26]. Such policies serve to exclude those who are most marginalized and, arguably, most harmed by the legacies of traditional research [19, 57].

In what follows, we aim to provide rich, ethnographic accounts of our research encounters and processes, focusing on the occasionally fraught aspects of our work. While this article foregrounds the messiness of our partnership, there have also been many moments of cohesion and smooth collaboration. We have chosen to focus on mess not because it was the primary experience of our work together, but because critically reflecting on messiness in CBPR can be generative [12, 37, 58]. As Cook [12] argues, accounting for messiness in CBPR can build methodological rigour. Similarly, Thomas-Hughes [58] describes how accounting for mess in collaborative research with young women emboldened the team members to identify dynamics of power and obligation embedded in their work. In our experience, accounting for mess has generated the ethical and empirical insights that we present here.

## Methods of reflection

The impetus for this article arose during a YAC meeting in February 2020. Madison and Reith mentioned that other university researchers in their networks had asked them about their experiences with CBPR. This sparked a conversation about what we would tell other research teams who want to start a YAC. We decided to build on this conversation to create an abstract submission for an upcoming public health conference. After that conference was cancelled due to the COVID-19 pandemic, the team agreed to turn the abstract into a manuscript for publication, with Madison taking the lead on writing. The content of these early 2020 meetings form the foundation of the insights presented in this article. We also draw from materials gathered between October 2018 (our first YAC meeting) and August 2021, as we describe in more detail below.

Some of our meetings were audio-recorded, which allowed Madison to present the group with YAC members’ direct quotes about their experiences with CBPR. All members of the YAC are also participants in our qualitative research program, and have all signed consent forms regarding the creation of audio recordings during their interactions with the university researchers, including during YAC meetings. When we are working on a paper or presentation—as we were while discussing our experiences with CBPR in early 2020—we often audio-record our conversations as a form of data collection. The university researchers always obtain permission from all YAC members before turning on the audio recorder, despite having previously obtained signed consent forms.

As Madison turned our abstract into a manuscript, she also reviewed meeting agendas and notes we made together to identify and further analyze specific encounters that were relevant to our emerging insights. The writing and analysis proceeded iteratively. As Madison wrote drafts of the article, she circulated them to the team to elicit feedback that was incorporated into subsequent versions. Some new ideas were developed through this process, while others were abandoned. We continued iteratively writing and analyzing until all team members endorsed the content, analyses, and conclusions of the manuscript.

For sections of this article that mention specific YAC members, Madison met with those individuals one-on-one to discuss what would be included and excluded. Those YAC members had final say over what was represented in these sections. The only exceptions to this co-writing protocol were one former YAC member who we lost touch with over time (Jordan), and another former YAC member who was not interested in being involved in co-writing (Alanna). All YAC member names appearing in this article are pseudonyms.

While we strove to achieve a collaborative writing process, it was ultimately led by Madison. Further, Danya provided the most thorough feedback in shaping the final article. We acknowledge that this process is in tension with the principle of shared leadership that is so central to our partnership. To some extent, this is an inevitable consequence of creating an output that is tailored to an academic setting and audience. Manuscripts are just one of many kinds of outputs that our team has created since the YAC began in 2018. We have also produced community reports, podcast episodes,^3^ video episodes,^4^ and events for community audiences (one of which is described in more detail below). Some of these outputs are arguably better suited to centering the voices of YAC members. Nonetheless, our entire team remained committed to creating an academic article because we agreed that this was a valuable way to communicate our findings to others who do collaborative work with young people who use(d) drugs.

## Introducing our team and the Youth Advisory Council (YAC)

In the fall of 2018, the university researchers set out to establish a CBPR project with young people who use(d) drugs that would explore their experiences with care across time and place. This project is embedded within a larger, 13-year program of anthropological and critical qualitative health research led by Danya since early 2008. Anthropological methods can share similarities with participatory research: both can be based on developing long-term, meaningful relationships with research participants, who over time become active collaborators [46]. Prior to 2018, Danya had typically collaborated with young people on a one-on-one basis. In 2018, she received funding via a CBPR funding stream that allowed her to expand this collaborative work to include a larger group of 8–12 young people and formalize aspects of it into a YAC. While the YAC emerged out of one CBPR research grant, it has since come to inform all of the work that we do.

Due to the size and ongoing, longitudinal nature of our research program, multiple research activities occur simultaneously. This allows for flexibility in terms of what projects YAC members choose to take on. Since our first meeting in October 2018, YAC members have helped to develop grant proposals, interview guides, and the various research outputs mentioned above. In 2018 and 2019, they undertook the lead role in planning and hosting a youth summit event focused on responses to the overdose crisis^5^ in Greater Vancouver. The summit included a ‘chill space’ where participants could use substances during the event under the supervision of trained overdose responders, making our event one of the few examples of a youth-dedicated supervised consumption site in the province [51]. Most recently, YAC members developed and delivered an online ‘how to work with youth’ workshop for undergraduate medical students. Our team’s work has been deeply enriched by the YAC, and conducting research activities without them has become virtually unimaginable.

YAC members are diverse in terms of age, race, gender identity, sexual orientation, and ability. They also share many experiences and outcomes of systemic marginalization. All YAC members have experienced periods of street-based homelessness and unstable housing in Greater Vancouver. They have also all navigated acute and chronic health problems in this setting, including overdoses and mental health crises. All YAC members have current or past experience with the intensive use of alcohol, stimulants (often crystal methamphetamine), and/or opioids (often fentanyl).

For most YAC members, experiences of housing instability, homelessness, health crises, and drug use extended into their childhoods. About half of YAC members were separated from their birth families by the state, and subsequently grew up moving between multiple government foster care and group homes before ‘aging out’ of most child welfare programs at age 19, and the remaining programs at age 25. For Indigenous YAC members, the trauma of these experiences is shaped by over a 100 years of racist policies aimed at the dispossession and assimilation of Indigenous peoples, including the Indian Act, the Potlatch Ban, the residential school system, and child welfare policies that forcibly removed thousands of Indigenous children from their families during the 1960s and 2000s (often known as the ‘60s and Millennium Scoops [22, 38, 49]). Indigenous young people continue to be vastly overrepresented in the child welfare system, demonstrating how the violence and harms of colonization extend into the present [5].

Across our collaboration, YAC members have critiqued how health research can lock participants into particular categories—including ‘Indigenous youth,’ ‘LGBTQ + youth,’ and even ‘young people who use(d) drugs.’ These categories are often constructed first and foremost in terms of ‘risk,’ ‘vulnerability,’ and ‘damage’ [56, 59]. While in many moments YAC members proudly identified themselves as Indigenous, LGBTQ+, and/or drug users, in other moments they foregrounded other forms of identification, positioning themselves as artists, employees, parents, romantic partners, activists, and persons in recovery (to list only some of the possibilities). It has been critical to our collaboration that we engage with YAC members on these different terms, creating space for multiple—and at times contradictory—forms of identification [59].

As we will demonstrate, the imperative to create this kind of space at times issued challenges to our guiding principles of shared leadership, safety, and inclusion. We define shared leadership as resisting tokenism by ensuring our processes are guided equally by young people alongside university researchers; safety as promoting and protecting the physical, psychological, and cultural well-being of all team members; and inclusion as minimizing barriers to participation for young people of diverse abilities.

We focus on these three principles because they were the ones that emerged as most important during our analytic process outlined above. When reflecting on the past 3 years, it became clear that how we understood and practiced shared leadership, safety, and inclusion was essential to characterizing our partnership—including moments when things did not go according to plan. We do not intend to suggest that these were our only guiding principles, or that they should always be the most important in guiding CBPR with young people who use(d) drugs (for more thorough explorations of CBPR guiding principles, see [14, 24]). In the following sections, we demonstrate the complexities inherent in putting these principles into practice, and the ethical and empirical advancements that can be generated through messy CBPR encounters. First, we introduce the setting in which these encounters occurred.

## Setting the scene

YAC meetings were held at our frontline research office until March 2020, when we transitioned to online meetings in response to the COVID-19 pandemic. In this article, we focus on describing our in-person meetings because these earlier meetings were most formative in developing our collective process. Our frontline research office is located in the Downtown South of Vancouver, a busy commercial, entertainment, and residential district. Processes of gentrification and poverty management continue to rapidly transform this part of the city [21]. Our research office shares a block with a liquor store, high-end sushi restaurant, Community Policing Centre, hair salon, and government-subsidized supportive housing building. Within a few blocks’ radius, interspersed among glassy condominiums and trendy coffee shops, are three ‘street youth’ drop-in centers, two busy community health centers, and Vancouver’s large, inner-city hospital.

Our office has an informal, non-institutional feel by design. The front door opens onto a small drop-in and waiting area, which we re-arrange for YAC meetings. We put large sheets of chart paper on the wall for notetaking, which hang alongside artwork created by young people and dozens of flyers advertising other research studies, local youth drop-ins, and free meal programs. A tall bookshelf filled with colourful public health brochures gets pushed back so we can squeeze a few more mismatched chairs in a circle around the room. In the adjacent bathroom, we make sure there is an empty sharps container for safe needle disposal. We stock up the cabinet by the front door with harm reduction supplies (e.g., glass pipes with vinyl mouthpieces for safer smoking, and sterile syringes, tourniquets, and alcohol swabs for safer injecting) so that YAC members can access these discretely. We ensure that naloxone overdose antidote kits are easily accessible, which all researchers and most YAC members have been trained to administer in the event of an overdose. The table that usually serves as a coffee and snack station for research participants is transformed into a buffet station with pizza, soda, candy, and a platter of cut vegetables and dip (YAC members insist on having one ‘healthy option’ at every meeting, although it rarely gets eaten). Finally, the university researchers ensure that they have two bus tickets and $30 cash with which to compensate each YAC member at the conclusion of the meeting (for a discussion on compensation in CBPR with people who use(d) drugs, see [45]).

Despite how cramped the drop-in and waiting area can become during YAC meetings, YAC members have consistently described it as a space where they feel at ease. This sense of comfort and ownership is shaped by a couple of factors. First, most YAC members have been coming to our office for years as research participants and were already familiar with the space and the university researchers when our collaboration began. Second, four YAC members also work as Peer Research Associates for another study run out of the office. These four individuals move through the office with an authority that signals to others that this is a space where young people are trusted and valued staff members.

A typical YAC meeting lasts between one and a half and two hours, with a break halfway through to stretch and go outside to smoke. For the first 10 or 15 minutes of the meeting, we eat and chat until everyone arrives. Our meetings begin with a round of ‘check-ins’—an opportunity for each member to share whatever is on their mind. The university researchers then propose a one or two item meeting agenda for YAC members to accept, reject, or expand on.

Initially, the university researchers feared that preparing an agenda would over-determine the activities of the YAC and undermine our principle of shared leadership. During our first few meetings, they did not set any agenda items, imagining that discussions and activities would simply emerge once we were all in the same room. However, after our second meeting several YAC members informed the university researchers that they experienced this approach (or lack thereof) as chaotic, unclear, and unproductive.

Eventually, we settled into a meeting routine that is quite flexible despite the university researchers preparing the agenda. Some meetings are highly social and fun, with significant time devoted to catching up with one another. Others are quiet and lethargic, during which everyone seems preoccupied with something else and all conversation fizzles out quickly. Still others are purposeful and productive, with YAC members insisting our conversations remain focused on achieving the tasks at hand. The university researchers adapt the agenda accordingly, often abandoning or adding agenda items on the fly to accommodate the different energies and priorities YAC members bring to each meeting. We have come to understand that setting the pace of our work together is an important means through which YAC members exercise leadership [46]. As we discuss in the following sections, the question of who should set the agenda of our meetings represented the first of several instances that forced the university researchers to reconsider their assumptions about what our guiding principles should look like in practice.

## Promoting shared leadership

### Balancing process and outcomes

For our team, promoting shared leadership begins with the physical space where YAC meetings take place. Holding meetings in a space where YAC members feel a sense of ownership engendered shared leadership from the outset of our partnership. However, as we have already mentioned, other aspects of shared leadership were less clearly established at the outset.

The question of who should set the meeting agenda demonstrates that the university researchers were initially most focused on the *process* of running a YAC (i.e., who runs our meetings) rather than on *outcomes* (i.e., what we accomplish at meetings). However, YAC members have repeatedly stressed that promoting shared leadership is not just about process, but must include a parallel commitment to producing tangible research outputs and meaningful change. YAC members do not ‘just want to sit around talking,’ no matter how youth-engaged the conversation is. As Kendra explained, ‘We want to, like, actually *do* something. A lot of times [in participatory projects] stuff gets brought up over and over again, but nothing happens.’

Respecting young people’s knowledge, expertise, time, and energy required the university researchers to recognize that process and outcomes cannot be thought of separately. Rather, our process needed to include a genuine commitment to moving research projects forward and creating change. YAC members have identified both smaller outputs (e.g., co-writing consent forms) and larger outputs (e.g., securing grant funding, co-writing articles, and producing podcast or video episodes) as meaningful. As Ocean reflected:

When [being part of a YAC] is good, you see change, you see things get funding that you wanted to get funding, and you see the world open its eyes to things you wanted to open its eyes to. As draining as it can be to spill all your shit on the table, you know in the end it’s really worth it. But when we can’t get anything done? Then we’re just spilling our shit on the table for, like, literally no reason.

### Learning and unlearning

In certain moments, promoting shared leadership has also been complicated by YAC members’ uncertainties regarding sharing their expertise and developing new skills. Some members have been part of tokenizing youth engagement projects in the past, and had lingering concerns about whether their input would be taken seriously. Others have institutional histories that involved being repeatedly ignored and degraded by various workers, providers, and other professionals—including researchers. As a result, some YAC members admitted that they needed to ‘relearn’ how to marshal confidence in this kind of collaboration.

For example, after spending many months designing activities for the youth summit, the university researchers suggested the YAC members could be the ones to facilitate these activities at the event. Given the amount of work YAC members had put into the project and the excellent facilitation skills many had demonstrated at our meetings, the university researchers were confident and excited about the idea of a youth-led summit, and they assumed the YAC members would be too. However, their suggestion was met with an awkward silence. Tentatively, a few YAC members explained that they were interested in leading the summit but needed help to prepare. Others said nothing at the time, but later admitted they were suspicious of whether the event would really be as youth-led as the university researchers were promising.

Together, we decided to meet more frequently in the months before the event to undertake facilitation training and practice summits. This extended preparation process gave YAC members time to develop new skills and confidence, and assuaged their concerns about being tokenized at the event. In the end, all team members agreed the youth-led summit was a huge success; it was well-attended, those who participated gave us positive feedback, and many rich conversations were had. The YAC members did an excellent job facilitating the event, and we have since decided to hold similar events in the future.

### Re-imagining shared leadership

In sum, the tensions and misunderstandings we encountered regarding shared leadership generated new understandings of how to enact that principle. We have re-imagined a youth-engaged process as one that requires university researchers to take the lead in certain moments, appreciates the significance of outcomes, and consistently affirms YAC members’ existing expertise while also supporting the development of new skills and confidence. In turn, this amplified our team’s capacity to take on novel projects like the summit.

## Fostering safety

### Triggering conversations

Fostering the physical, psychological, and cultural safety of all team members has been a top priority throughout our partnership. This commitment is evidenced by practices we have already mentioned, like having harm reduction supplies and naloxone kits available, beginning each meeting with a round of check-ins, and scheduling breaks into each meeting. And yet, at our fourth YAC meeting we were confronted with the complexities of maintaining a safe space for all young people, and the contradictions that can arise between promoting safety and promoting shared leadership.

At the time, we were developing a qualitative, semi-structured interview guide about methadone, buprenorphine-naloxone, and other forms of opioid agonist therapy (OAT). The university researchers asked the YAC members to start by reflecting on their own experiences with OAT. Alanna got the conversation started, emphatically relating how going on buprenorphine-naloxone had ‘saved her life.' Kendra, who had been on methadone for several years, whole-heartedly disagreed. She described how difficult it can be to stop taking OAT once starting it, and how she felt ‘handcuffed’ by the requirements of regular doctor’s appointments and daily witnessed doses at a pharmacy, as well as the addictive properties of the pharmacotherapy itself.

After several minutes of heated discussion, Jordan tentatively revealed that he had been thinking about starting OAT, but given what others had just said, he was now less sure about trying it. Jordan’s sudden reluctance was alarming to those of us who viewed OAT as potentially life-saving in the context of the current overdose crisis. Danya quickly reminded the group that what is true for one person might not be true for another, and attempted to suggest to Jordan that OAT might be worth trying despite the negative experiences of individuals like Kendra. The conversation continued, but Jordan did not say anything else about OAT during that meeting. A couple of weeks later, we lost contact with Jordan when he moved to Manitoba to be with family. It is not possible for us to know what impact—if any—that YAC meeting had on Jordan’s decision-making surrounding OAT. Nonetheless, many of us continued to worry about how conversations during YAC meetings could impact individuals’ willingness to access potentially life-saving forms of care.

Towards the end of the meeting, a different challenge to fostering safety arose. Thorn became frustrated by the entire conversation, arguing that all of the focus on OAT was distracting us from what they viewed as the ‘real solution' to the overdose crisis: drug decriminalization and a greater focus on youth-specific harm reduction, including youth-dedicated safer consumption sites. Building on Thorn’s critique of medicalized approaches to drug use and addiction, Raven then mentioned a set of animal-based studies which are collectively known as ‘the Rat Park Study’ [2, 31]. Very briefly, these studies placed rats in different living conditions, and found that rats consumed less morphine water when they lived in enriched environments compared to isolated cages. After summarizing the research, Raven shared their interpretation of the findings: ‘That *proves* we don’t need medications,’ they said confidently. ‘All we need to do is give people better environments.’

At the mention of the Rat Park Study Thorn abruptly stood up, said they needed a smoke, and went outside. At the end of the meeting, Thorn pulled Danya aside to explain that they had been deeply offended and ‘triggered’ by Raven’s mention of an animal-based study as an explanation for why people use drugs. ‘People who use drugs should not be compared to rats,’ they stated emphatically. Danya asked Thorn if they wanted her to address the issue with the group, but they asked her not to. A week later, Madison and Reith saw Thorn at our frontline research office for an appointment. During the conversation, it became clear that Thorn’s Indigenous identity shaped how they had been impacted by Raven’s comment. Thorn reflected, ‘I’ve been thinking about racism a lot lately. Like when people talk about that Rat Park Study. A study with rats can’t acknowledge racism.’

Raven, who is Métis, did not view the Rat Park Study as neglecting the history and ongoing effects of racism and colonization. By mentioning Rat Park, they intended to move our conversation beyond medicalized approaches to addiction (like OAT) to foreground how the environments in which people live shape substance use and related harms. Nevertheless, Thorn’s commitment to the politics of humanizing people who use drugs—especially other Indigenous young people who use drugs—meant that for them, mentioning an animal-based study was offensive and unacceptable.

### Re-imagining safety and trauma-informed practice

In Vancouver’s service and research landscape, ‘trauma-informed’ is a phrase often used to signal a best practice for engaging with young people who use(d) drugs, and a commitment to fostering safety in these engagements. A trauma-informed approach can be defined as maintaining constant awareness of how past trauma experienced by individuals shapes their emotional reactions and behaviors in the present [7]. A trauma-informed approach also involves fostering trust and choice, which are essential to creating a space that prioritizes non-violence and avoids replicating past traumatic experiences [7]. In our work, this has looked like allowing YAC members to take breaks whenever they need to, and assuring them that our collaboration will continue even when things become difficult.

We are all highly committed to trauma-informed practice and safety. However, we have found that promoting an atmosphere that centers the diverse positionalities, perspectives, and convictions of YAC members means it is virtually impossible to avoid difficult and triggering conversations. Our discussions can be unsafe in various ways, including triggering feelings of dehumanization and racism (as Thorn experienced) and desires to avoid or disengage from services (as Jordan experienced). These triggers can have serious and lasting consequences. At the same time, preventing these consequences would require the university researchers to control YAC meetings rather than sharing leadership, and curtailing how YAC members’ are able to express diverse positionalities, commitments, experiences, and knowledges at our meetings.

YAC members have openly critiqued this kind of silencing, which occurs in many youth-focused treatment and recovery settings. In these places, young people are often expressly forbidden to talk about their experiences with substance use and crime. While these ‘anti-trigger’ policies are ostensibly meant to promote safety, YAC members have said that they don’t experience them that way. Instead, such silencing can undermine the trust and choice that is essential to trauma-informed practice, including the choice to work through difficult experiences with other young people.

Overall, the messy moments surrounding safety forced our team to recognize the limits of this principle. While trauma-informed practice guidelines gave us a beneficial starting point, we have since developed a more nuanced and flexible understanding of what it means to foster safety.

## Upholding inclusion

### Diverse participation

From the outset of our collaboration, our team has been committed to minimizing the barriers that young people who use(d) drugs often face when trying to participate in CBPR projects. For example, we do not have any rules about YAC members’ substance use, how many meetings they should attend, or what their participation should look like.

Everyone contributes to our meetings in different ways. Some of us, like Alanna, Thorn, and Danya, are comfortable speaking up about a wide range of topics and are often the ones to get our conversations started. In contrast, Stan and Reith are more comfortable sitting back, waiting for topics that they have direct experience with. Kendra and Madison like to have printed materials during meetings so that they can make notes in the margins, sometimes exchanging those notes with each other afterwards. Some YAC members come to nearly every meeting without fail, while others come and go depending on their abilities and interests. Making space for these different forms of participation is a crucial aspect of inclusivity for our team.

Furthermore, shifts in YAC members’ broader life circumstances often impact how they contribute to the YAC. Since our partnership began, nearly every YAC member has had to manage multiple personal crises, including periods of unstable mental health, hospitalizations, unsafe housing situations and homelessness, conflicts with family members and romantic partners, aging out of youth services, and grief as more friends and loved ones are lost to the ongoing overdose crisis. Each YAC member has navigated these crises differently in terms of their participation in meetings. Some stopped attending meetings during these periods of time, and inclusivity looked like allowing them to come back whenever they were ready, regardless of how long they had been away, without requiring them to justify their absence. Others continued to attend meetings, and their difficulties—as well as corresponding changes to their behavior—became disruptive to team activities. As we explore in the rest of this section, simultaneously maintaining inclusivity, safety, and shared leadership in these instances became challenging, and at times, impossible.

### Inclusion during periods of crisis

In the summer of 2019, Kat and her girlfriend of several years broke up. In the following weeks, Kat’s mental health declined and her stimulant use intensified. Kat was typically one of the quieter members of our group, generally preferring to share insights with the university researchers after or outside of YAC meetings. However, after her breakup, Kat began speaking up frequently, sometimes interrupting other YAC members and often taking our conversations in confusing directions. Kat also became less discrete about her stimulant use. While we have no rules regarding intoxication at YAC meetings, YAC members generally expect each other to be discrete about their substance use to avoid triggering other members who are trying to reduce or remain abstinent from drug use.

After 3 or 4 months, several YAC members became frustrated with Kat’s behavior, lamenting that our meetings had become unproductive. We were preparing for the summit at the time, and as we have already mentioned, many of us were anxious about the preparation needed for the event. Then, Kat stopped by Thorn’s supportive housing building without warning in the middle of the night to ask them for harm reduction supplies. This raised concerns for many of us about violations of personal boundaries. Some YAC members said it was no longer appropriate for Kat to attend meetings.

However, the university researchers were unwilling to ask Kat to leave the group. This decision was shaped by their commitment to inclusivity, and an awareness that asking a young person to leave the YAC during a period of crisis would replicate the punitive responses youth often encounter in service settings (responses we frequently critiqued during YAC meetings). In addition, the university researchers worried that asking Kat to leave the YAC, even temporarily, could further destabilize her during a period of significant vulnerability. While we all agreed on the importance of upholding inclusion, the situation with Kat revealed that many of us had different understandings of what this should look like in practice. Some YAC members argued that inclusivity should not come at the price of productivity. Others, while frustrated with Kat’s behaviour, understood periods of crisis as an inevitable part of our work, and did not necessarily agree that Kat should be asked to leave the group.

Rather than ask Kat to leave the YAC, the university researchers tried strategies like taking more breaks during meetings, inviting a community nurse to attend as a source of additional support, and splitting into smaller groups so that discussions were easier to facilitate. These changes improved group dynamics, but did not entirely ameliorate them. After a few months of disrupted productivity, some YAC members stopped attending meetings. At that point, it had become obvious that our commitment to inclusivity and our commitment to shared leadership were pitted against each other, with no resolution in sight.

After several months of intense stimulant use and episodes of mania, Kat entered a period of equally intense depression. During this time, she was once again quiet at meetings, and while her mental health crisis continued, it was less disruptive to our activities. While YAC meetings were largely ‘back to normal,’ many of us remained deeply concerned about how to best help Kat.

In the spring of 2020, Kat decided to enter residential drug treatment. At the time of writing, she recently moved from a second-stage recovery house into her own studio apartment, and celebrated 18 months of abstinence from substance use. She continues to stay in close contact with Madison and Reith, although she no longer attends YAC meetings. ‘[The YAC] was an important support for me at the time,’ Kat reflected with Madison and Reith over coffee one day. ‘But it was also a really rough time in my life, and now I want to leave it behind.’

### Unresolved dilemmas as ethical practice

Unlike our other stories about the messiness of enacting shared leadership and safety, our experiences with Kat did not necessarily lead us to a new understanding of what inclusion means for our team. In making YAC meetings inclusive for Kat, we perhaps undermined inclusivity and safety for other young people, as well as the focus on producing tangible outcomes that we learned are essential to promoting shared leadership. And yet, the YAC was one of Kat’s only supports at the time, and it is entirely possible that her crisis could have been worse—and in the context of a contaminated drug supply, even deadly—had she been asked to leave the YAC. We continue to feel conflicted about whether there may have been better ways to support both Kat and the other YAC members during that time.

In sum, despite our team’s shared commitment to certain guiding principles, our understandings of how those principles should be applied varies because of the diverse positionalities, life circumstances, perspectives, and convictions of each person on our team. This means that for us, decision-making by consensus is not always an option. We have come to understand that honouring YAC members’ lived and living experiences means acknowledging that some disagreements may never be fully resolved.

## Conclusion: The generative potential of mess

As we reflect on our work together, we recognize that our commitment to shared leadership, safety, and inclusion has been central to the success of our collaboration. These principles have shaped our decision-making about the location, structure, and pace of our meetings, and the expectations that we enforce and resist when working together. However, there have been several moments when enacting shared leadership, safety, and inclusion became rife with ‘complexities and contradictions’ [8]. Many of these encounters, while fraught, have also been ethically generative, leading us to re-imagine shared leadership and safety in more nuanced and flexible ways. When these principles came into conflict with our commitment to inclusivity, however, there was no resolution to be found (see also [53]). We came to understand that making space for unresolved dilemmas—rather than trying to manufacture consensus—can also be part of ethical practice. We join others in arguing that expecting CBPR partnerships to operate based on consensus perhaps erases, or at least undermines, the diversity that exists within these collaborations [35, 62].

Much of the messiness we have encountered across our collaboration can be traced to the diverse and shifting positionalities, life circumstances, perspectives, and convictions of each person on our team. Avery Gordon [29] refers to this as ‘complex personhood.’ Recognizing YAC members’ complex personhoods is essential to resisting the essentialization and erasure of young people who use(d) drugs, and has therefore been necessary to the ethics of our CBPR project. It is a means of moving beyond a view that positions young people who use drugs as primarily ‘vulnerable’ or a ‘problem’ to be ‘fixed,’ and towards one that foregrounds their complicated priorities and desires [59].

Further, complex personhood acknowledges that all individuals ‘are beset by contradiction’ ([29], p. 4). In our own work, there have been many examples of such contradictions, like when YAC members simultaneously demanded meaningful control in research processes *and* expressed doubts about their abilities to take on leadership roles, or when they critiqued policies that punish young people during periods of crisis *and* chastised the university researchers for refusing to ask a person in crisis to leave the YAC. The contradictions and multiple desires expressed by YAC members have complicated our understandings and enactments of shared leadership, safety, and inclusion. But we have come to embrace this messiness and its generative potential [12, 37, 58]. Part of CBPR’s value is that it engages directly with subjugated knowledges that university researchers do not typically have access to [10]. Efforts to avoid or minimize messiness can flatten not only young people’s complex personhoods, but also the empirical insights that can be generated  through CBPR collaborations. In our case, moments of tension generated new analyses about how young people navigate substance use, OAT, racism, mental health crises, and research participation across time and place [27, 48]. The push-back from Thorn and Raven at our meeting focused on OAT, for example, led us to develop an interview guide that focuses equally on harm reduction and housing alongside OAT, which has led to richer interviews with research participants.

Our findings build on previous work demonstrating that the practice of CBPR with young people must create space for moments of conflict and change [37, 53, 58]. Models of participation must be flexible, dynamic, and attuned to the personal, social, cultural, and structural contexts that shape participatory projects [14, 40]. Cahill and Dadvand [9] evoke the imagery of machinery and inter-connecting gears to present their 7-P framework for engaging in participatory research with youth, drawing attention to how purpose, positioning, perspectives, power relations, protection, place, and process interconnect—albeit not always smoothly. Similarly, Arunkamar and colleagues [3] present a ‘rope ladder’ model of youth participation that accounts for flexibility and responses to exogenous forces. When CBPR projects fail to make space for mess, they contribute to a trend in which institutions invite participation only when the form of that participation is acceptable to institutional norms [14]. This qualified form of participation severely limits the knowledge and ethics that CBPR can generate.

Conceptualizing the messiness of CBPR as generative also has implications for how CBPR is evaluated. Previous work has identified the systematic evaluation of CBPR as a priority for the field’s continued development [10, 64]. Our reflections suggest that evaluations of CBPR may be problematic if they employ principles like shared leadership, safety, and inclusion as straightforward evaluation metrics. When putting these principles into action leads to complexity, contradiction, and a downright mess, it does not necessarily mean that something was done wrong. Instead, it may be that this mess is ethically necessary and empirically valuable to the practice of CBPR in some contexts.

Finally, the insights we have gained through messy CBPR have been personally and politically important for each of us. For example, Stan explained to Madison that being a YAC member has led him to confirm his ‘mini-conspiracies’ about ‘how the world works.’ By this, he meant that collaborating with other young people who use(d) drugs—even when it was difficult—has allowed him to connect his own experiences of marginalization to broader social, structural, and historical injustices. This new understanding has shaped how Stan thinks about himself and the social changes he wants to fight for. Similarly, Ocean described how marginalization and oppression can become normalized for young people whose lives are marked by poverty, housing instability, and substance use. ‘But at YAC meetings,’ she reflected, ‘we fight our normal. We tell each other that we deserve better.’

## Data Availability

To protect young people’s confidentiality, data used for this article will not be made publicly available.

## Footnotes

1 We recognize that some YAC members fall outside of standard age range definitions of “youth” (e.g., “youth” as referring to those between 14 and 24 years of age). However, we retain the use of this term here and in several other places to signal how most YAC members were regarded institutionally throughout our collaboration, as well as how all members tended to view themselves. It is interesting to note that, in many moments, older YAC members strongly asserted their continued inclusion in the “youth” category, and frequently distinguished themselves from populations of older people who use(d) drugs in Vancouver.

2 The land now known as Greater Vancouver is located on the traditional, unceded, and continuously occupied territories of the Coast Salish Peoples, including the Sḵwx̱wú7mesh (Squamish), səl̓ilwətaɁɬ (Tsleil-Waututh), and xʷməθkʷəy̓əm (Musqueam) First Nations. We use the name Vancouver in this article because it is the most commonly recognized name for this place, which continues to be produced by colonization and structural and everyday racism.

3 Available at: https://crackdownpod.com/podcast/episode-28-after-the-flood/.

4 Available at: https://youtu.be/Fn2S7Re7oCQ.

5 In 2016, a public health emergency was declared in the province of British Columbia in response to a dramatic increase in overdose deaths, driven largely by the proliferation of illicitly-produced fentanyl and its analogues in local, criminalized (and therefore unregulated) drug markets. Over 1700 young people ages 10–29 have lost their lives to overdose since 2016 [6].
